# Balloon-Assisted Percutaneous Thrombin Injection for Treatment of Iatrogenic Left Subclavian Artery Pseudoaneurysm in a Critically Ill COVID-19 Patient

**DOI:** 10.1155/2021/4245484

**Published:** 2021-10-07

**Authors:** Hassan Al-Thani, Ahmed Hussein, Ahmed Sadek, Ali Barah, Ayman El-Menyar

**Affiliations:** ^1^Vascular Surgery Section, Department of Surgery, Hamad General Hospital (HGH), Doha, Qatar; ^2^Radiology Department, HGH, Doha, Qatar; ^3^Clinical Research, Trauma and Vascular Surgery Section, HGH, Doha, Qatar; ^4^Clinical Medicine, Weill Cornell Medical College, Doha, Qatar

## Abstract

**Background:**

Central venous catheter represents an important tool in the management of critically ill patient. In this report, we described a COVID-19-positive case who had COVID-related complications and iatrogenic left subclavian artery pseudoaneurysm after central venous catheter insertion. *Case Presentation*. A 58-year-old male patient presented with a high-grade fever, myalgia, and shortness of breath due to COVID-19 infection. He required mechanical ventilation support and hemodialysis. He also developed uneventful deep vein thrombosis and myocardial infarction. As a complication of central line insertion, the patient developed pseudoaneurysm that originated from the subclavian artery with significant bleeding and large hematoma. Balloon-assisted percutaneous thrombin injection was done under ultrasound guidance. The patient was extubated 2 days later with no evidence of flow in the pseudoaneurysm. However, he lost movement in the left arm secondary to the compression of the brachial plexus from the pseudoaneurysm/hematoma, and therefore, 1.5 litres of the hematoma was evacuated in the operating room through a lateral left chest wall incision along the anterior axillary line to relieve the compression over the brachial plexus. The patient declined surgical reconstruction of the brachial plexus, and the flaccid paralysis of the arm did not recover during the follow-up.

**Conclusion:**

This is a case of unusual complications of COVID infection and iatrogenic left subclavian artery pseudoaneurysm postcentral vein cannulation. Balloon-assisted percutaneous thrombin injection for treatment of left subclavian artery pseudoaneurysm is feasible; however, delayed diagnosis could be associated with long-term or permanent disability.

## 1. Introduction

Central venous catheter represents an important tool in the management of critically ill patients [1]. During the peak of the COVID pandemic, more critically ill patients required admission into intensive care unit with increased number of central venous catheter insertion with possible increase in the rate of complications [2] including the rare ones as subclavian artery pseudoaneurysm. In this report, we described a case of COVID-related complications and iatrogenic left subclavian artery pseudoaneurysm after central venous catheter insertion in a patient with severe COVID-19 infection.

## 2. Case Report

**Figure 1:**
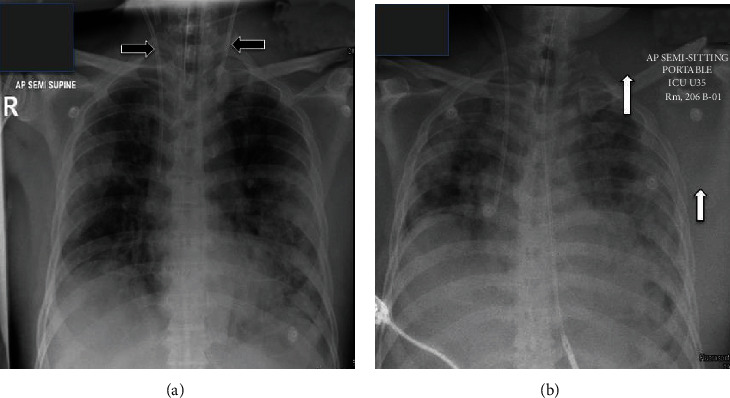
(a) Chest X-ray showing two double lumen hemodialysis central vascular access. (b) Chest X-ray after the removal of the one on the left side with significant soft tissue swelling around the left shoulder associated with left lateral displacement of the scapula.

A 58-year-old male patient presented with high-grade fever, myalgia, and shortness of breath and proved to be COVID-19 positive on the 16^th^ of May 2020. The patient's clinical condition rapidly deteriorated on the second day of admission due to severe pneumonia that required mechanical ventilation support after failure of the conventional support including prone position. He was started on azithromycin 500 mg intravenous, tocilizumab 600 mg intravenous single dose, hydroxychloroquine 400 mg tablets once a day, and methylprednisolone 40 mg twice daily (for five days for all). He developed acute kidney injury requiring renal replacement therapy in the form of sustained low-efficiency dialysis (SLED) through a right femoral vein central line due to hemodynamic instability on the 19^th^ of May. The patient developed deep vein thrombosis on the same side of the hemodialysis catheter despite the prophylaxis with low molecular heparin. A second central line using the left internal jugular was placed under ultrasound guidance on the 24^th^ of May. When the patient was connected to the hemodialysis machine, the venous pressure was high which indicates possible arterial flow. Another line was inserted on the 25^th^ of May on the right internal jugular using ultrasound guidance, and at this time, it worked well (Figure 1(a)). The left internal jugular catheter was removed, and pressure dressing was applied (Figure 1(b)).

The patient developed left upper arm and supraclavicular swelling, and his hemoglobin dropped by 4-5 to 5 g from the initial 9.5 g. The patient was stabilized by fluid resuscitation and blood transfusion. No melena or any sign of active bleeding was identified. The patient was extubated on the 1^st^ of June, breathing well with retrosternal chest pain with elevated troponin level which was diagnosed as myocardial infarction type II that was treated conservatively. He also had left shoulder pain and was unable to move his left arm; orthopedic consultation was requested and the plan for conservative management as no obvious radiological orthopedic insult.

**Figure 2:**
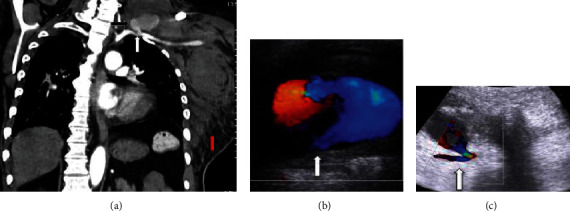
(a) CT coronal section showing the left subclavian artery (white arrow) with pseudoaneurysm (black arrow) and large intra- and extramuscular chest wall and left side hematoma (red arrow). (b, c) Ultrasonography showing a pseudoaneurysm originating from the subclavian artery (white arrow).

**Figure 3:**
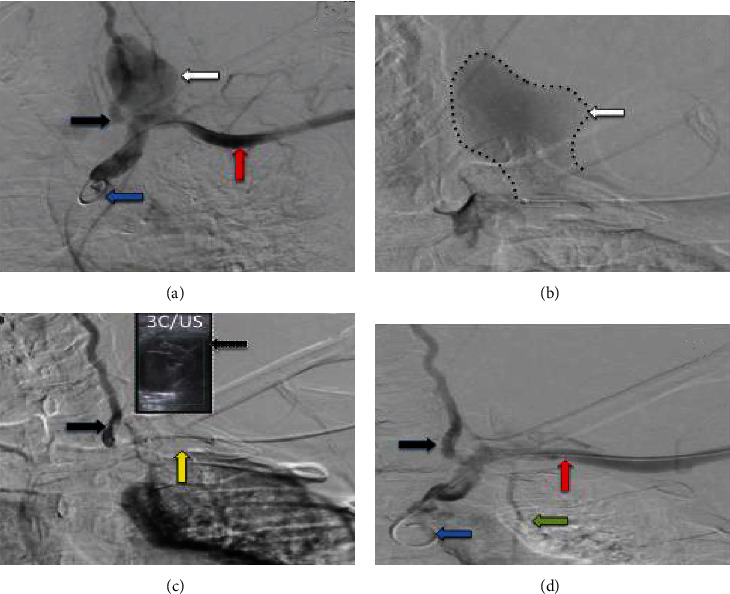
(a, b) Pigtail catheter (blue arrow) arteriogram showed pseudoaneurysm (white arrow) arising from the proximal part of the left subclavian artery (red arrow) distal to the origin of the left vertebral artery (black arrow). (c) Angioplasty balloon-tipped catheter (yellow arrow) covering the neck of the pseudoaneurysm. Bedside ultrasound documents the absence of the flow within the pseudoaneurysm by inflating the balloon, and injection of thrombin was done under ultrasound guidance ((c) and US). (d) Arteriogram after the thrombin injection shows no pseudoaneurysm, and it shows the left subclavian artery (red arrow), left vertebral artery (black arrow), and left internal mammary artery (green arrow).

A CT scan of the abdomen and upper chest was done to rule out the source of bleeding on the 2^nd^ of June. CT scan confirmed evidence of large intramuscular and extramuscular hematoma involving the muscle of the left side of the chest wall extending to the lateral aspect of the abdominal wall, and there was no intra-abdominal free fluid or collection. The CT scan was limited to the upper chest and abdomen but did not cover the neck and the supraclavicular area. There was a plan for another CT scan of the neck and upper chest, but because of acute kidney injury and risk of more nephrotoxicity, the CT scan was postponed until the 9^th^ of June when his kidney function improved and his last hemodialysis was on the 7^th^ of June. CT angiogram for the head, neck, and chest revealed a large homogenously hyperdense sac-like structure with a tongue projecting superiorly noted posterior to the left clavicle and in close proximity to the anterior wall of the proximal part of the left subclavian artery possibly arising from it with narrow neck, measuring 2.1 × 3.5 × 4.5 cm (Figure 2). It was seen associated and surrounded with large mixed density area extending from the left lower neck opposite the level of epiglottis towards the left axilla, subscapular region, lateral chest wall, and the left upper abdominal quadrant approximately more than 25 cm in craniocaudal dimension and 8 cm in maximum transverse dimension in the lateral chest wall with more heterogeneous density inferiorly. The rest of the left subclavian artery appeared stretched by the hematoma but otherwise patent. The left internal jugular vein was significantly attenuated and could not be seen in the lower neck. Ultrasound showed pseudoaneurysm originating from the subclavian artery (Figures 2(b) and 3(c)).

Vascular surgery consultation was done on the 12^th^ of June regarding the large hematoma extending from the neck to the left side of the chest. Attempt to evacuate the pseudoaneurysm was postponed because of the fear of rupturing the pseudoaneurysm. The left arm was flaccid with no movement; however, pulses over the brachial and radial arteries were palpable with no sign of ischemia.

The patient was transferred from the COVID facility to the tertiary hospital for management of the pseudoaneurysm on the 13^th^ of June with full infection control precautions taken, as the patient's COVID test remained positive for 6 weeks. Due to his general condition and extent of the hematoma, the plan was to use ultrasound-guided thrombin injection of the pseudoaneurysm. This was done under general anaesthesia to reduce the risk of coronavirus transmission in case of the need to proceed with open surgical repair for the pseudoaneurysm. On the 15^th^ of June, under ultrasound guidance with complete aseptic technique and local anaesthesia, micropuncture set was used to puncture the left brachial artery. Using Seldinger technique, access of the aortic arch with a 6-French sheath was obtained. Flush aortic angiogram was done using a 4-French pigtail catheter; it showed pseudoaneurysm arising from the proximal part of left subclavian artery distal to the origin of left vertebral artery and left internal mammary artery (Figures 3 and 3(b)). Mapping of the neck of pseudoaneurysm was done, and an 8 mm × 3 cm balloon was used to cover the pseudoaneurysm neck. Bedside ultrasound documented the absence of the flow within the pseudoaneurysm by inflating the balloon (Figure 3(c)). After inflation of the balloon, injection of 2000 U of thrombin was done under ultrasound guidance (Figure 3(c)). Follow-up left subclavian angiogram showed good opacification of the left vertebral artery and internal mammary artery with nonopacification of the pseudoaneurysm (Figure 3(d)). The sheath was removed, and manual compression for 10 minutes was applied at the puncture site. Postcompression ultrasound of the left brachial artery showed good flow distally with no hematoma. Radial and ulnar pulses were intact.

**Figure 4:**
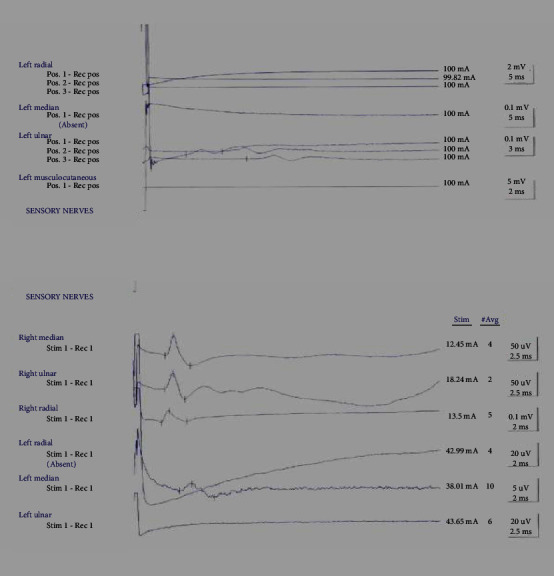
Nerve conduction studies of the upper limbs.

The patient was extubated 2 days after the procedure; however, he had no movement in the left arm which was considered secondary to the compression of the brachial plexuses from the hematoma which was confirmed by electromyography (EMG) and nerve conduction studies (Figure 4). On the 24^th^ of June, the patient was taken to the operating theatre for evacuation of 1.5 litres of hematoma through a lateral left chest wall incision along the anterior axillary line to relieve the compression over the brachial plexus. The patient was offered surgical reconstructions for the brachial plexus (grafting and transfer) but he declined. Despite that, the patient's flaccid paralysis did not recover during the three months of follow-up.

## 3. Discussion

The present COVID-positive case developed many complications related to the COVID infection such as reversible organ failure (lung and kidney) and arterial and venous thromboembolism (myocardial infarction and deep vein thrombosis) as well as iatrogenic complication (subclavian pseudoaneurysm and nerve injury). For faster detection of such iatrogenic injuries in an intubated patient, the healthcare giver should follow proper physical examination, bedside imaging, and CTA imaging with little contrast when renal failure is present, in addition to adherence to the precautionary measures during pandemic. Cannulation of the central veins can have various complications (4-35%) including pneumothorax, chylothorax, carotid artery injury, stroke, arteriovenous fistula, pseudoaneurysm, nerve injury, cardiac perforation, and cardiac tamponade [3]. Although some of these complications are rare, it can be fatal if not discovered and treated successfully and on time [4]. Pseudoaneurysm formation is diagnosed based on clinical findings of pulsatile mass, palpable thrill, or pressure and ischemic manifestations although it might be asymptomatic. Imaging modalities include ultrasonography, CT angiography, MR angiography, and conventional angiography [5].

In our case, the investigations were focused on finding the cause of dropping of hemoglobin with the big hematoma in the chest wall extending into the upper abdomen which caused delay in the diagnosis and treatment of the left subclavian pseudoaneurysm. The conventional CT and CT angiogram offered valuable information regarding the anatomy and morphology of the pseudoaneurysm and helped in planning the treatment options. Symptomatic pseudoaneurysm should be treated; traditionally, it was treated by surgical repair which carries significantly higher morbidities and mortality rates.

The management of the subclavian artery pseudoaneurysm evolved over the years. Although open surgical repair had been the primary method in its management, due to the difficult surgical exposure and the difficulties associated with proximal and distal control, other less-invasive approaches have been utilized, including endovascular option. Endovascular approach is aimed at excluding the pseudoaneurysm from the circulation by covered stent or by embolization. On the other hand, percutaneous approach usually utilizes ultrasound or CT-guided thrombin injection. Direct compression of the pseudoaneurysm using ultrasound guidance is another noninvasive management. This needs a cycle of 10-20 minutes or up to 1 hour with operator and patient discomfort. There are limitations for its use including location and depth and the outcome affected using anticoagulants, size, configuration, and diameter of the neck of the pseudoaneurysm. The treatment options should be tailored to the site, rupture risk, clinical situation, and patient comorbidities [6].

In our case, the suitable size of the covered stent was not available. Our choice was to proceed for thrombin injection to induce thrombosis of the pseudoaneurysm and exclude it from the circulation. This technique was developed initially for the treatment of the femoral pseudoaneurysm after catheterization and extended to other vessels [7].

Few case reports described thethrombin injection technique for the treatment of the subclavian artery pseudoaneurysm [4, 7–16]. The success rate is around 82% and can be used as a salvage procedure of failed endovascular stenting with low complication rate. In our case, we successfully used the balloon-assisted percutaneous injection of thrombin for the treatment of left subclavian artery pseudoaneurysm in the critically ill COVID-19 patient.

Lastly, the fear of direct contact and lack of proper daily physical examination of patients with severe COVID infection could lead to serious outcomes because of missing important signs particularly in intubated patients who had invasive procedure such as center line insertion. There are few case reports in the literature describing the subclavian aneurysm causing brachial plexus injury even after removal of the subclavian central line with a partial recovery [3]. Also, subclavian artery pseudoaneurysm has been reported to cause brachial plexus injury [17, 18]. The authors stated that early surgical intervention of compressive hematoma within the first 2 days showed improvement in all patients; however, late intervention > 2 days could lead to improvement in only 50% of cases [17].

## 4. Conclusion

These are unusual complications in a COVID ill patient. Iatrogenic subclavian artery injury and pseudoaneurysm secondary to central vein cannulation are not common but could be associated with serious morbidity. Balloon-assisted percutaneous thrombin injection without stenting for treatment of left subclavian artery pseudoaneurysm is feasible; however, delayed diagnosis and evacuation of the subsequent hematoma could be associated with long-term or permanent disability.
